# 

**Published:** 1887-09

**Authors:** 


					﻿SEPTEMBER, 1S&7-
OLD
VoL pv"
xP®L,s^
& 1855 6
iJD'TTj
NEW SERIES
V0LIV.-N0. 7.V
Wm»
lEDIGAL-'^URGO
B J0UBNAK85
^^^X^WESTMOREtANO®
H.V.W41LLER.M.D.LL.D.1
J *m	'fJAMES A.GRAY, M.D."
i k I \	. nl
Published Byjames p.harrison&cq
iflk	jST LANTA, GA.
SUBSCRIPTIONS 12.50 A YEAR. "
				

